# 下呼吸道及肺癌内菌群与肺癌发生发展的关系及其临床应用

**DOI:** 10.3779/j.issn.1009-3419.2023.101.33

**Published:** 2023-12-20

**Authors:** Bowen LI, Zhicheng HUANG, Yadong WANG, Jianchao XUE, Yankai XIA, Yuan XU, Huaxia YANG, Naixin LIANG, Shanqing LI

**Affiliations:** ^1^100730 北京，中国医学科学院，北京协和医学院，北京协和医院胸外科（李博文，黄志诚，王亚东，薛剑超，夏研凯，徐源，梁乃新，李单青）; ^1^Department of Thoracic Surgery,Peking Union Medical College Hospital, Chinese Academy of Medical Sciences (CAMS) and Peking Union Medical College (PUMC), Beijing 100730, China; ^2^100730 北京，中国医学科学院，北京协和医学院，北京协和医院风湿免疫科 （杨华夏）; ^2^Department of Rheumatology and Clinical Immunology, Peking Union Medical College Hospital, Chinese Academy of Medical Sciences (CAMS) and Peking Union Medical College (PUMC), Beijing 100730, China

**Keywords:** 肺肿瘤, 呼吸系统, 细菌, Lung neoplasms, Respiratory system, Bacteria

## Abstract

由于16S rRNA测序技术的发展，长期以来被认为是不存在的下呼吸道菌群也逐步被揭示出来。这些微生物与疾病（如肿瘤）的相关性是近年来的研究热点。由于肿瘤周围环境中的菌群会进入肿瘤内部，研究者们也开始关注肿瘤内菌群的生物学行为以及其与肿瘤的相互作用。在这篇综述中，我们展示了下呼吸道菌群的特点，并总结了近年来关于这些菌群与肺癌关系的相关研究成果。同时，我们也对肿瘤内菌群的基本特征进行整理，并着重阐述肺癌内菌群的特点及其与肺癌发生、发展的关系。最后，我们综述了下呼吸道和肺癌内菌群在临床上的转化应用方向，并汇总了样本采集、测序和控制污染的要点，希望为肿瘤的早筛和治疗提供新的思路。

## Abstract (en)

Due to the advancement of 16S rRNA sequencing technology, the lower respiratory tract microbiota, which was considered non-existent, has been revealed. The correlation between these microorganisms and diseases such as tumor has been a hot topic in recent years. As the bacteria in the surrounding can infiltrate the tumors, researchers have also begun to pay attention to the biological behavior of tumor bacteria and their interaction with tumors. In this review, we present the characteristic of the lower respiratory tract bacteria and summarize recent research findings on the relationship between these microbiota and lung cancer. On top of that, we also summarize the basic feature of bacteria in tumors and focus on the characteristic of the bacteria in lung cancer. The relationship between bacteria in lung cancer and tumor development is also been discussed. Finally, we review the potential clinical applications of bacterial communities in the lower respiratory tract and lung cancer, and summarize key points of sample collection, sequencing, and contamination control, hoping to provide new ideas for the screening and treatment of tumors.

由于既往对微生物的检测依赖于微生物培养，健康人的下呼吸道曾经被认为是无菌的。然而，即使在最恶劣的自然条件下也发现了微生物的存在，与口腔及外界环境相沟通的下呼吸道中没有细菌显然不合情理。2010年，Hilty等^[1]^第一次利用支气管镜和16S rRNA测序技术证明了下呼吸道细菌的存在，并展示了哮喘、慢性阻塞性肺疾病患者和健康人下呼吸道菌群的构成差异，开启了对下呼吸道菌群的研究。由于近年来研究肠道菌群的作用在肿瘤免疫治疗领域取得了引人注目的成就，一些研究开始着眼于呼吸道菌群对于肺癌发生、发展和治疗的影响。同时，在泛癌种中针对肿瘤内菌群的研究^[2]^又一次引发了人们对于微生物与肿瘤之间关系的思考。鉴于肿瘤内微生物与其所处的器官环境中的微生物种群高度相关却又存在差异，本文将立足于呼吸道菌群和与其息息相关的肺癌内菌群，总结二者的特点和在肺癌发生、发展中的作用，并展示其临床应用前景。此外，本文也将整理目前肺部菌群研究中样本收集、测序和污染控制的要点，从而更好地评估各研究结论中可能出现的假阳性及假阴性结果，体现微生物测序在临床样本中应用的进展和局限性。

## 1 下呼吸道菌群的特点

在下呼吸道中，细菌数量自上而下逐渐递减；相比于上呼吸道，其细菌负荷低100-10,000倍，肺内每平方厘米可检测到约2200个细菌基因组，每克肺组织有10^3^-10^5^个细菌^[3⇓⇓⇓-7]^。这种低负荷缘于下呼吸道更深的解剖位置、黏膜-纤毛清洁机制、低营养环境以及肺内的表面活性剂和抑菌液层^[8]^。下呼吸道菌群的构成与上呼吸道一致，因此可以推测其主要来源是上呼吸道的微吸入物质，主要的细菌种群为拟杆菌门（Bacteroidetes）、厚壁菌门（Firmicutes）、变形菌门（Proteobacteria）和放线菌门（Actinobacteria）^[3,8]^。普雷沃氏菌属（Prevotella）、链球菌属（Streptococcus）、韦荣氏球菌属（Veillonella）、奈瑟菌属（Neisseria）、嗜血杆菌属（Haemophilus）和梭杆菌属（Fusobacterium）是健康人肺内丰度最高的菌属^[5]^。肺内菌群的具体构成会因被采样者的生活环境、生活习惯、气候、环境污染水平甚至饲养宠物类别而异，因此各研究不具有统一结论^[9]^。

影响肺内微生物动态平衡有3个因素：迁入、清除、不同菌种的相对增殖速度（往往由局部生长条件决定）^[10]^。这3个因素的改变会引起下呼吸道短暂或持续的菌群失调，如胃食管功能障碍或反流会增加细菌的迁入，吸烟或慢性支气管炎导致黏膜清除细菌的能力降低，炎症反应导致的局部生长条件改变可能会造成该区域菌种差异性增殖。肺部疾病是影响菌群失调的最重要的始动原因，气道的炎症导致黏液、血浆渗出增加，从而制造出营养更丰富、温度更高和缺氧的微环境；同时炎性细胞因子还会促进特定菌种生长，活化的免疫细胞对各菌种也有不一致的杀伤效果。这些效应最终造成了菌群的选择性生长，而这些生长的菌群会通过其病原相关分子模式（pathogen-associated molecular pattern, PAMP）和代谢物进一步促进炎症反应^[10]^。这种自我放大的正反馈回路可能导致呼吸道微环境的永久改变以及微生物的永久性紊乱。

## 2 下呼吸道菌群与肺癌的关系

相对于健康人，肺癌患者下呼吸道菌群的丰富度降低，优势属为Prevotella（20%）、Streptococcus（12%）和Veillonella（8%）^[11,12]^。非小细胞肺癌（non-small cell lung cancer, NSCLC）和小细胞肺癌（small cell lung cancer, SCLC）的下呼吸道菌群之间存在显著的组成差异。晚期肺癌患者下呼吸道菌群比早期患者更接近于口腔的菌群组成^[4,12]^。同样，生存不佳的患者相比于长生存期患者，其下呼吸道样本微生物的构成也与口腔样本更相似。上、下呼吸道微生物组成越相似，进展的可能性越大，这种紊乱菌群的特征与预后之间存在的关系可能与白细胞介素17（interleukin 17, IL-17）、磷脂酰肌醇3-激酶（phosphatidylinositide 3-kinase, PI3K）、有丝分裂原活化蛋白激酶（mitogen-activated protein kinase, MAPK）和细胞外调节蛋白激酶（extracellular regulated protein kinase, ERK）等通路上调有关^[4]^。

下呼吸道菌群紊乱与肿瘤发展的因果关系也在探索中。紊乱的下呼吸道菌群可能通过其对炎症-增殖信号的激活、与免疫细胞的相互作用以及代谢物或毒素促进肿瘤的发生和发展^[13]^。暴露于Veillonella、Prevotella和Streptococcus的A549细胞系ERK和PI3K通路显著上调^[13]^。细菌的配体会上调IL-1β和IL-23的水平，激活并扩增Th17和γδT细胞，诱导炎症出现并促进肿瘤发生^[14]^。在KP肿瘤小鼠模型中，Veillonella的引入使小鼠生存降低、体重减轻、肿瘤负荷增加，同时肿瘤内招募了更多的Th17细胞和中性粒细胞，IL-17水平升高^[4]^。而使用抗生素或益生菌[鼠李糖乳杆菌（Lactobacillus rhamnosus GG）或双歧杆菌（Bifidobacterium）]雾化吸入来改变肺内免疫抑制性微环境，可以显著降低小鼠肿瘤细胞静脉注射后成瘤的数量，并可以增加抗肿瘤药物治疗的疗效^[15]^。因此，下呼吸道菌群具有促进和抑制肿瘤发生、发展的双面作用。肺微生物组既可以抵抗致病性病原的增殖，也可以在肺免疫抑制性微环境的形成中起到重要作用^[15]^。

## 3 肿瘤内细菌和肺癌内细菌的特点

关于肿瘤内细菌特点的研究往往在泛癌种中进行，近年来也开始关注肺癌（表1）。癌症基因组图谱计划（The Cancer Genome Atlas Program, TCGA）数据库的6.4×10^12^条测序的短读序列中，7.2%为非人类数据；在这些非人类数据中35.2%为细菌、古菌或病毒的遗传序列^[16]^。肿瘤内细菌丰度很低，仅0.1%-10%肿瘤细胞中存在细菌^[17]^。16S rRNA测序结果表明肿瘤内既存在革兰氏染色阳性（G+）菌，也存在革兰氏染色阴性（G-）菌，主要以胞内菌形式存在于肿瘤细胞和免疫细胞内，且一般定位在细胞质中^[2]^。在肺癌中，癌细胞内的细菌负荷最高，显著高于各种免疫细胞和基质细胞内^[18]^。胞内菌可能改变自己的包装方式，进化为细胞壁缺乏型细菌（类似于L型细菌），具有高度可变的大小和形状^[2]^。

**表1:** 肺癌内菌群相关研究及主要发现

Authors, Year	Sample size	Main findings
Yu G, et al. 2016^[24]^	31 (lung tumor)+ 31 (non-malignant lung tissue)	1. α diversity was significantly higher in non-malignant lung tissue than in tumor tissue. 2. Compared with squamous cell carcinoma, tissues of adenocarcinoma had increased relative abundance ofThermus and decreased relative abundance ofRalstonia.
Greathouse KL, et al. 2018^[21]^	1255 (lung tumor)+ 1256 (non-tumor adjacent tissue) (from databases)	1. There were distinct microbial populations within squamous cell carcinoma and adenocarcinoma. 2. Among squamous cell carcinoma, Acidovorax was more abundant in smokers and patients with TP53mutation.
Peters BA,et al. 2019^[20]^	19 (lung tumor)+ 19 (remote normal lung tissue)	1. Lung cancer tissue had lower richness and diversity than, but compositionally similar to paired normal lung tissue. 2. Families Koribacteraceae and Lachnospiraceae in lung cancer tissue were associated with reduced RFS and DFS.
Nejman D,et al. 2020^[2]^	245 (lung tumor)+ 231 (normal tissue)	1. The bacterial load of lung cancer was at an intermediate level among pan-cancer. 2. Compared with other tumor types, metabolic pathways that degrade chemicals in cigarettes were enriched in the bacterial community of lung cancer. 3. Several metabolic pathways related to the biosynthesis of metabolites that can be used by plants were enriched in bacteria of lung cancer of smokers.
Wong LM,et al. 2020^[39]^	930 (lung tumor)+ 930 (adjacent normal tissue) (from databases)	Age and gender were important factors affecting the bacterial composition in lung cancer.
Patnaik SK,et al. 2020^[19]^	48 (lung tumor)+ 48 (adjacent non-tumor lung tissue)	Due to the low abundance of bacteria in tumors and adjacent tissue, the results were susceptible to contamination and the authors did not compare the bacteria in these two types of samples.
Leblond N,et al. 2021^[22]^	29 (lung tumor)+ 29 (healthy adjacent tissue)	Enteric, potentially pathogenic and pro-inflammatory bacteria were more frequently found in cancer than healthy tissue.
Leng Q, et al. 2021^[40]^	31 (lung tumor)+ 31 (noncancerous lung tissue)	Compared with noncancerous lung tissues,Acidovorax was overrepresented in squamous cell carcinoma tissues and Capnocytophaga DNA was enriched in adenocarcinoma tissues.
Rolle A, et al. 2022^[18]^	12 (lung tumor)+ 12 (adjacent normal region)	1. In tumor microenvironment, bacterial burden was significantly higher in tumor cells compared with other types of cells. 2. Genes involved in the Wnt/β-catenin, hypoxia, and angiogenesis pathways showed a strong positive correlation with bacterial burden.

RFS: recurrence-free survival; DFS: disease-free survival.

一般来说，肿瘤内菌群的生物多样性低于其对应的正常组织，这暗示肿瘤可能会因为其独特的微环境选择性扩展某些细菌种群^[17]^。在进行肺癌组织与癌旁正常肺组织的菌群丰富度和多样性比较时，各研究^[18⇓-20]^的结论不一致。肿瘤内的菌群主要来源于其所处的器官。肺癌组织或癌旁肺组织中菌群多样性显著低于唾液和支气管灌洗液样本，这些肺癌内部的细菌可能来源于附近的小气道^[19]^。同时这些菌群也可能来自于血液循环系统（如一些来自于肠道的细菌），例如相比于正常肺组织，肺癌组织中存在更多肠道细菌和一些潜在病原体^[21,22]^。肿瘤的血管系统高度混乱、内皮连接不完整以及部分血管为盲端血管的特点导致了其高渗透性和血流缓慢的特征，加之肿瘤内免疫抑制的微环境，使肿瘤可能会为血液循环中的细菌提供庇护所^[23]^。

不同癌种，甚至是不同基因型或表型肿瘤中的细菌组成也有所不同。肺腺癌中的细菌负荷高于肺鳞癌，瘤内菌群组成也不一致^[18]^。如肺腺癌中栖热菌属（Thermus）相对丰度比肺鳞癌更高，而罗尔斯通菌属（Ralstonia）和噬酸菌属（Acidovorax）更低^[21,24]^。在肺鳞癌中，Acidovorax在吸烟者或TP53突变患者中丰度更高^[21]^。

肿瘤所处的微环境影响其内部微生物种群的组成以及富集代谢通路。吸烟患者NSCLC病灶中的细菌显著富集了一些降解香烟中化学物质的代谢通路，以及一些植物代谢通路（可能来源于烟草中的植物相关细菌）^[2]^。不吸烟者肺癌中的细菌负荷高于吸烟者，可能是因为吸烟塑造了局部的抗菌环境^[18]^。

肿瘤内菌群不仅是肿瘤发生、发展的“果”，也起到促进肿瘤进展的“因”的作用^[17]^。肿瘤内菌群或菌体成分可以调节肿瘤细胞的干性、上皮-间充质转化、黏附程序和机械压力反应程序促进肿瘤的发展^[25⇓⇓⇓-29]^。CTNNB1（涉及Wnt/β-catenin通路）、HIF1A（涉及缺氧）和VEGFA（涉及血管生成）等基因表达水平与肺癌内细菌负荷呈正相关；而TP73（涉及细胞周期）和TLR5（涉及模式识别）与肺癌内菌群负荷呈负相关。一些涉及上皮-间充质转化的基因表达水平与肺癌内细菌负荷存在相关关系，暗示肺癌内细菌可能有促进肿瘤转移的能力^[18]^。此外，被细菌感染肿瘤细胞可以通过外泌体激活其他细胞的Wnt/β-catenin等通路，因此没有被细菌寄生的肿瘤细胞也能受到细菌的影响^[30]^。

## 4 下呼吸道菌群/肺癌内菌群在临床上的应用方向

### 4.1 复发风险分层与生存预测

肺癌患者正常肺组织中更高的细菌丰富度和多样性、更高丰度的毛螺菌科（Lachnospiraceae）、粪杆菌属（Faecalibacterium）、瘤胃球菌属（Ruminococcus）和罗斯氏菌（Roseburia）与更差的预后相关，而更高丰度的科里氏菌科（Koribacteraceae）却预示着更好的预后^[20]^。

在I期肺癌患者中，复发患者术前唾液中代尔夫特菌属（Delftia）丰度更高而Bifidobacterium丰度更低、肿瘤组织中葡萄球菌属（Staphylococcus）更多而芽孢杆菌属（Bacillus）和厌氧芽胞杆菌属（Anaerobacillus）更少。利用术前支气管肺泡灌洗液对呼吸道菌群进行评估是预测早期肺癌术后复发风险的合适样本形式^[19]^。而IIIB-IV期肺癌患者上、下呼吸道微生物组成越相似，进展的可能性越大。相比于完全缓解（complete response, CR）或部分缓解（partial response, PR）的患者，肿瘤进展患者的下呼吸道菌群富集Veillonella、Streptococcus、Prevotella和罗氏菌属（Rothia）^[4]^。

### 4.2 肿瘤治疗

目前很少有下呼吸道菌群与免疫治疗效果相关的研究，但在肠道菌群领域的相关研究可以提供思路。2018年，Routy等^[31]^发现抗生素影响免疫检查点抑制剂（immune checkpoint inhibitors, ICIs）的临床获益，对ICIs响应和不响应的晚期NSCLC患者的肠道细菌组成具有差异。肠道无菌荷瘤小鼠在移植对ICIs响应者的粪便微生物后可以增强对ICIs的敏感性^[31⇓-33]^。

肺内菌群与肠道菌群有很多相似处，例如二者都与外界环境沟通且存在黏膜免疫系统^[34]^。有些学者^[35]^提出“肠-肺”轴的假设，希望能将两个微生物组进行联系。事实上，已经发现有证据^[9]^提示肠、肺微生物组可能具有直接或间接的交互作用。两个微生物组之间的直接交互在于在解剖结构上二者便有相互沟通的可能性。肺内的自然清扫机制可以将肺内细菌排入肠道，而胃内容物的微吸入则有可能使肠道细菌进入肺内。同时，肠道菌群可以通过PAMPs调节免疫细胞分化、活化、分泌细胞因子，这些细胞或物质可以通过黏膜淋巴管和血液循环影响到呼吸道黏膜，间接影响肺黏膜的炎症环境与菌群构成^[8]^。

对ICIs响应者和无响应者的肠菌对比研究^[8]^的结果有两个共性：响应者肠道细菌多样性更高，以及响应者肠道菌以厚壁菌为主导。这些对ICIs响应的肿瘤内体现为CD8^+^ T细胞浸润增加、骨髓来源抑制性细胞（myeloid derived suppressor cells, MDSCs）浸润减少和调节性T细胞（regulatory T cells, Tregs）浸润减少。有趣的是，Le等^[15]^发现利用雾化吸入抗生素来改变下呼吸道菌群组成的小鼠的肺转移瘤数量显著减少，且肿瘤内产生IL-10的Tregs减少、自然杀伤（natural killer, NK）细胞活性增强。小鼠雾化吸入Lactobacillus rhamnosus GG后，树突状细胞（dendritic cells, DC）和NK细胞活性上调，M2相关基因Il10、Ido和Irf4表达下调，转化生长因子-β（transforming growth factor β, TGF-β）和前列腺素E2（prostaglandin E2, PGE2）表达有下调趋势；而M1相关标志IL-12和干扰素调节因子5（interferon regulatory factor 5, IRF5）显著上调。雾化吸入Lactobacillus rhamnosus GG或Bifidobacterium还可以显著增强抗肿瘤药物的作用。这些结果提示了与肠道菌群相似，肺内菌群也有希望成为未来增强抗肿瘤治疗的辅助靶点。

### 4.3 液体活检

血液样本中的血液微生物DNA（blood-based microbial DNA, mbDNA）可以提供肿瘤的信息，识别早期肿瘤，并可以实现将一种癌种与其他癌种进行区分。基于数据库中泛癌种患者血液和肿瘤组织中微生物组的基因信息，可以通过检测mbDNA将肺癌患者与健康人或其他癌种（如前列腺癌）患者鉴别出来^[16]^。

## 5 研究下呼吸道菌群/肺癌内菌群的相关技术

### 5.1 样本采集

常选取的标本形式有口腔刷取组织、支气管刷取组织、支气管肺泡灌洗液、手术切除样本和呼出气冷凝物^[36]^。

有研究^[37]^在探索肺支气管菌群与肺癌的关系时提出应该选择肿瘤另一侧肺的支气管进行取样，以减少肿瘤堵塞导致下呼吸道微生物变化的影响；也有研究^[11]^在邻近肿瘤的段支气管中取材。在取癌旁正常组织作为肿瘤内菌群对照时，应注意尽量靠近肿瘤取材，避免因支气管等级不相同而带来的固有的菌群差异。

### 5.2 测序技术

16S rRNA测序是目前应用最广泛的研究下呼吸道/肺癌内菌群的检测方法。16S rRNA片段包含9个高变区（V1-V9）以及在细菌中高度保守的区域。其中，V4区域特异性好，是进行细菌注释和多样性分析的最佳选择^[38]^。而Nejman等^[2]^对16S rRNA基因上的5个区域进行扩增和测序，相对于一般的V4或V3-V4扩增检测方法提高了覆盖范围和分辨度。

### 5.3 污染控制

避免污染是研究肿瘤菌群的关键思想。由于下呼吸道内和肿瘤内的微生物含量极少，轻微的污染即可导致显著的差异性结果。应建立较为全面的无菌取样流程，尽量避免实验室前污染，如从待检测样本石蜡块边缘取纯石蜡进行对照测序。同时应避免实验室污染，在DNA提取和聚合酶链式反应（polymerase chain reaction, PCR）扩增过程中设置阴性对照^[2]^。最后，对数据进行恰当处理也能有效减少污染的干扰。

## 6 总结

由于肿瘤周边及肿瘤内存在独特的解剖学、生理学和免疫学环境，细菌需要发展出与肿瘤微环境相匹配的功能学特征，形成了独特的微生物种群。这些独特的微生物又通过活化各种促炎及促增殖的信号通路影响肿瘤的发生和发展。目前的研究多关注下呼吸道菌群/肺癌内菌群在肺癌患者和健康人、肺癌组织和正常肺组织之间的差异，然而各研究结果并没有得到统一的差异菌群，这是因为下呼吸道菌群/肺癌内菌群受到地区环境、空气污染以及个体遗传等条件的影响较大。因此相对于试图描绘出具体的差异细菌种系，更好的方向是聚焦于这些差异细菌富集的功能及代谢通路是否具有一致性，从功能学的角度来分析肿瘤内细菌对环境的适应和其可能对肿瘤发展造成的影响。

从转化医学的角度上说，肺癌内独特细菌种群可以将自己的DNA释放入血液系统，基于具有全血样本基因信息的数据库，可以对肺癌患者进行早期鉴别，对于缺少关键基因突变的肺癌尤其具有意义。在肺癌的治疗中，通过雾化吸入抗生素或细菌，可以便捷并有效地调控呼吸道菌群的分布，而且不会轻易改变全身菌群尤其是肠道菌群的组成，有效提高治疗效果。此外，检测下呼吸道中以Veillonella为代表的口腔定殖菌群可以预测肺癌患者复发或进展的风险。下呼吸道菌群在免疫治疗疗效的预测上更是具有前景。然而，由于下呼吸道菌群/肺癌内菌群极低的丰富度，污染对于肺部菌群研究和临床应用是致命的，如何科学地取样以及控制测序过程中的污染将是肺部菌群研究的关键。

**Competing interests**

The authors declare that they have no competing interests.
